# Could Increased Expression of Hsp27, an “Anti-Inflammatory” Chaperone, Contribute to the Monocyte-Derived Dendritic Cell Bias towards Tolerance Induction in Breast Cancer Patients?

**DOI:** 10.1155/2019/8346930

**Published:** 2019-11-18

**Authors:** Ana Paula Silva de Azevedo-Santos, Mirtes Castelo Branco Rocha, Sulayne Janayna Araujo Guimarães, André Alvares Marques Vale, Fabio Martins Laginha, Flavia Raquel F. Nascimento, Maria Aparecida Nagai, Patrícia C. Bergami-Santos, José Alexandre Marzagão Barbuto

**Affiliations:** ^1^Biological and Health Sciences Center, Federal University of Maranhão, Avenida dos Portugueses, 1966, Bacanga, São Luis, Brazil; ^2^Pérola Byington Hospital, Avenida Brigadeiro Luis Antonio, 683 São Paulo, Brazil; ^3^The State of São Paulo Cancer Institute (ICESP), Avenida Dr. Arnaldo, 251 São Paulo, Brazil; ^4^Institute of Biomedical Sciences, University of São Paulo, Avenida Prof. Lineu Prestes, 1730 São Paulo, Brazil

## Abstract

Dendritic cells (DCs) are the most efficient *antigen*-*presenting cells* and link the innate immune sensing of the environment to the initiation of adaptive immune responses, which may be directed to either acceptance or elimination of the recognized antigen. In cancer patients, though DCs would be expected to present tumor antigens to T lymphocytes and induce tumor-eliminating responses, this is frequently not the case. The complex tumor microenvironment subverts the immune response, blocks some effector mechanisms, and drives others to support tumor growth. Chronic inflammation in a tumor microenvironment is believed to contribute to the induction of such regulatory/tolerogenic response. Among the various mediators of the modulatory switch in chronic inflammation is the “antidanger signal” chaperone, heat shock protein 27 (Hsp27), that has been described, interestingly, to be associated with cell migration and drug resistance of breast cancer cells. Thus, here, we investigated the expression of Hsp27 during the differentiation of monocyte-derived DCs (Mo-DCs) from healthy donors and breast cancer patients and evaluated their surface phenotype, cytokine secretion pattern, and lymphostimulatory activity. Surface phenotype and lymphocyte proliferation were evaluated by flow cytometry, interferon- (IFN-) *γ*, and interleukin- (IL-) 10 secretion, by ELISA and Hsp27 expression, by quantitative polymerase chain reaction (qPCR). Mo-DCs from cancer patients presented decreased expression of DC maturation markers, decreased ability to induce allogeneic lymphocyte proliferation, and increased IL-10 secretion. In coculture with breast cancer cell lines, healthy donors' Mo-DCs showed phenotype changes similar to those found in patients' cells. Interestingly, patients' monocytes expressed less GM-CSF and IL-4 receptors than healthy donors' monocytes and Hsp27 expression was significantly *higher* in patients' Mo-DCs (and in tumor samples). Both phenomena could contribute to the phenotypic bias of breast cancer patients' Mo-DCs and might prove potential targets for the development of new immunotherapeutic approaches for breast cancer.

## 1. Introduction

Dendritic cells (DCs) are mononuclear phagocytes, specialized in antigen presentation to naïve T cells and, consequently, to initiation and control of immunity in immunogenic or tolerogenic response [1–3]. In cancer context, DCs are crucial for the induction of a potent immune response; on the other hand, defects in their differentiation/maturation can be favorable to tumor escape [4]. The complex relationship between tumor cells and the host immune system is dynamic, and different stimuli can induce heterogeneous DC subsets [5, 6]. A tumor immunoenvironment presents chronic inflammation that contributes to cancer development and progression and increases the accumulation of myeloid-derived suppressor cells [7].

Tumor cells produce several factors that affect DC differentiation. Heat shock proteins (Hsps) are a chaperone protein family induced by cell stress. Hsps have antiapoptotic properties and are actively involved in tumor cell proliferation and invasion [8]. Small heat shock protein 27 (Hsp27) has a role in protection against toxicity mediated by inflammation conditions. Moreover, the expression of Hsp27 induces monocyte to produce IL-10, which is a strong inhibitor of the Th1 response and is constantly found to be elevated in human cancers [9–11].

Breast cancer is the most common invasive cancer in women; in this context, Hsp27 is associated with tumor growth regulation and drug resistance in human breast cancer [11–14]. Banerjee et al. demonstrated that the treatment of monocytes with Hsp27 leads to the differentiation for macrophages with a tolerogenic profile, being these similar to the macrophages found in breast tumors [15]. Laudanski et al. (2007) reported that exogenous inhibition of Hsp27 in monocytes leads to differentiation in immature dendritic cells, and its activation is associated with impaired antitumoral immune responses [10]. Taking into account this theoretical framework, our objective is to evaluate the phenotype and biological function of monocyte-derived DCs from patients with breast cancer as well as the role of Hsp27 in this process.

## 2. Materials and Methods

### 2.1. Subjects and Study Design

This was a prospective, single-blind study with convenience sampling, based on researcher availability of breast cancer patients undergoing mastectomy surgery. The protocol was approved by the National Commission of Ethics in Research (CONEP) (695/CEP) and was conducted in the Hospital Pérola Byington (107/06), São Paulo, Brazil. Samples were collected only after obtaining informed consent of donors. Peripheral blood mononuclear cells (PBMCs) were obtained from 18 female healthy volunteers (32 to 50 years) and 20 female patients (33 to 62 years). The histological diagnostics confirmed 14 ductal breast carcinomas, 4 lobular breast carcinomas, and 2 ductal and lobular breast carcinomas (pT_1-4_, pN_0-2_ and M_0_).

Initially, we obtained DCs derived from monocyte by *in vitro* culture with IL-4 and GM-CSF, adding TNF-*α* for DC maturation. The patients and healthy donors' Mo-DC phenotypes were characterized by flow cytometry and the functional activity by mixed lymphocyte reaction culture and cytokine secretion. Afterward, the Mo-DCs were cultured with or without breast cancer cell lines for the phenotype and functional characterization. The IL-4 and GM-CSF receptors were investigated in monocytes by flow cytometry. Tumor samples were used to evaluate the Hsp27 expression by quantitative polymerase chain reaction (PCR).

### 2.2. Mo-DC Culture

We followed the methods of Barbuto et al. [16]. PBMCs were separated over a Ficoll-Paque gradient (*d* = 1.076), resuspended, and seeded in 12-well plates in AIM-V medium. After overnight incubation at 37°C, nonadherent cells were removed, and the adherent cells were cultured in the presence of GM-CSF and IL-4 (50 ng/mL; R&D, Minneapolis, MN, USA) in AIM-V medium. On the 5th day, TNF-*α* (50 ng/mL; R&D, Minneapolis, MN, USA) was added for DC activation. After 2 further days in culture, the cells were harvested with cold RPMI 1640 and analyzed for flow cytometry. The culture efficiency was calculated for the percentile of cells removed starting from the total of adherent cells by well.

For tumor coculture in transwells, aliquots of tumorigenic (MCF7) and metastatic (SKBR-3) breast cancer cell lines (1 × 10^5^ in 100 *μ*L of medium) were pipetted (6.5 mm diameter, 0.4 *μ*m pore size polycarbonate transwell filters) (Corning B.V. Life Sciences, Schiphol-Rijk, The Netherlands) in Mo-DC culture (5^th^ day). After 7 days, the DCs were harvested with cold RPMI 1640 for phenotype and functional characterization.

### 2.3. Flow Cytometry of Immune Cell Populations

The monocytes and Mo-DCs were analyzed according to their size and granularity. To detect specific surface antigens, the immune cells (5 × 10^5^ cells) were stained with fluorescein isothiocyanate (FITC), phycoerythrin (PE), or PE-Cy5-conjugated mouse monoclonal antibodies (MoAbs) against HLA-DR, CD11c, CD86, CD116, and CD124 or with mouse isotype controls (Caltag Laboratories, Burlingame, CA, USA) and analyzed in a FACSCalibur cytometer (Becton Dickinson, San Jose, CA, USA) with Win MDI2.8 software. At least 10,000 gated events were acquired per antibody analyzed. The expression of the markers was determined for the number and median fluorescence intensity (MFI) of positive cells.

### 2.4. T lymphocyte Isolation by Rosetting with AET-Treated Sheep Erythrocytes and Allogenic T Cell Proliferation Assay

Nonadherent cells obtained previously after adherent assay of PBMC from healthy donors were incubated for 1 hour with S-(2-aminoethyl) isothiouronium bromide hydrobromide- (AET-) treated sheep red blood cells. T lymphocytes that adhered to red cells (R+), forming rosettes, were submitted a Ficoll gradient 900g for 35 min and isolated from the erythrocyte pellet by disaggregation and lysis of red blood cells with hypotonic saline solution (0.899% NH_4_Cl) for 2 min. After that, the cells were washed twice in RPMI 1640 supplemented with 10% heat-inactivated fetal calf serum (FCS) and 2 mM L-glutamine (R10 medium), centrifuged at 250g for 10 min for the removal of red cell *debris*. T lymphocytes were used in MLR (mix lymphocyte reaction) to evaluate Mo-DC ability to induce lymphoproliferation *in vitro*.

The ability of DCs from healthy donors and breast cancer patients to stimulate allogeneic T cells was assessed in this assay. The T cells used in all the experiments were collected from healthy volunteers and breast cancer patients. In this assay, DCs were the stimulator cells and T lymphocytes the responder cells. The latter were added at 5 × 10^4^ cells/well. In each assay, all tests were carried out using two replicates. Appropriate controls were set up in each 96-well plate (Costar, Cambridge, UK). The stimulator DCs were irradiated with 25 Gy. The T cells were stained with CFSE. DC to T cell ratio (1 : 30) was set up, and the plates were cultured for 7 days at 37°C in a humidified 5% CO_2_ incubator. The proliferation index was determined by FlowJo software. The results as a percentage (%) were calculated through the reason of control fluorescence mean/DCs treated to T cell culture fluorescence mean multiplied by 100 and divided by the reason of control fluorescence mean/DCs treated without T cell culture fluorescence mean. The culture supernatants were frozen for cytokine analyses.

### 2.5. Quantification of IFN-*γ* and IL-10 by ELISA

IFN-*γ* and IL-10 concentrations in lymphocyte-DC allogenic culture supernatants were tested using commercially available quantitative enzyme-linked immunosorbent assay (ELISA) kits (Becton Dickinson, San Jose, CA, USA).

### 2.6. Real-Time Quantitative Polymerase Chain Reaction

Total mRNA from the Mo-DC and tumor breast cancer cells was isolated using Illustra RNAspin Mini Isolation Kit (GE Healthcare, Piscataway, USA). The concentration of total mRNA was determined by measuring the absorbance at 260 nm using a GeneQuant pro (Amersham Biosciences, Cambridge, England). Reverse transcription was carried out with a real-time quantitative polymerase chain reaction, which contains DNAse I to avoid DNA contamination (Promega, Madison, EUA). mRNA Hsp27 expression was analyzed using Power SYBR Green master mix (Applied Biosystems, Warrington, UK) according to the manufacturer's instructions. The fold change in target gene expression between the various groups was determined using the comparative Ct (2^−*ΔΔ*Ct^) method, after normalizing to glyceraldehyde-3-phosphate dehydrogenase (GAPDH) and *β*-actin expression as an internal reference.

### 2.7. Statistical Analysis

Comparisons of the samples to establish statistical significance were determined by an unpaired *t*-test. Results were considered to be statistically significant when the *p* < 0.05. We used the GraphPad Prism 7 statistical program (OSB Software, São Paulo, Brazil) to analyze the results.

## 3. Results

**Figure 1:**
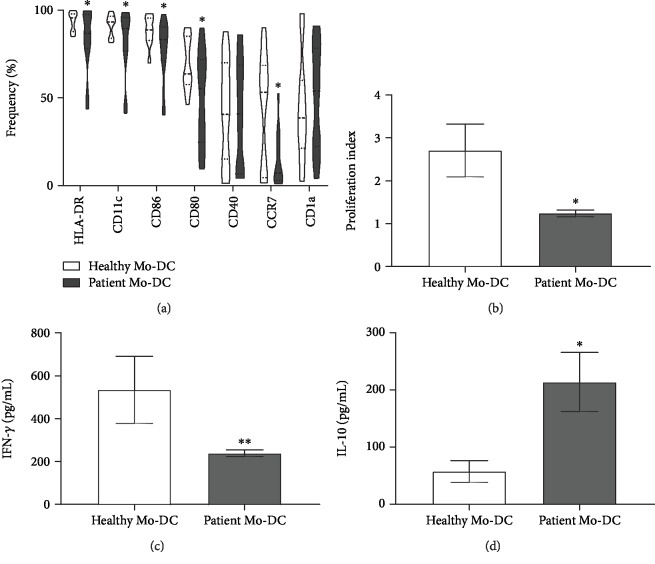
Mo-DCs from a breast cancer patient show lower frequency of maturation surface markers than those from healthy donors. (a) Box plots represent the frequency of DC maturation markers. Monocytes were cultivated for 7 days in the presence of GM-CSF and IL-4 (50 ng/ml each) to induce the differentiation of immature Mo-DCs; cell maturation was induced by TNF-*α* (50 ng/mL) during the last 2 days of culture. On day 7 of culture, Mo-DCs were harvested and labeled with monoclonal antibodies specific to the molecules CD1a, CD11c, CD80, CD86, CD40, HLA-DR, and CCR-7. (b) Allogeneic T lymphocytes previously labeled with CFSE were cocultured with Mo-DCs (from healthy donors or from patients). On day 4 of coculture, the cells were harvested, stained with a viability marker, acquired by flow cytometry, and an index of proliferation was determined by FlowJo software. (c, d) Concentration of IFN-*γ* and IL-10 in supernatants of cocultures with patients' Mo-DC or healthy donors' Mo-DC, determined by ELISA. The results are expressed as the average concentration (pg/mL) ± SEM. (*n* = 20 patients and *n* = 18 healthy donors); a *t*-test was used to compare patients and healthy donor groups. ^∗^*p* < 0.05.

Mature DC membrane markers were analyzed by flow cytometry, and Mo-DCs obtained from breast cancer patients showed less HLA-DR, CD11c CD86, CD80, and CCR7 expressions when compared to Mo-DCs from healthy donors (Figure 1(a)). Further, the patient's Mo-DCs had a lower lymphostimulatory capacity and secreted less IFN-*γ* and more IL-10 (Figures 1(c) and 1(d)) than controls.

**Figure 2:**
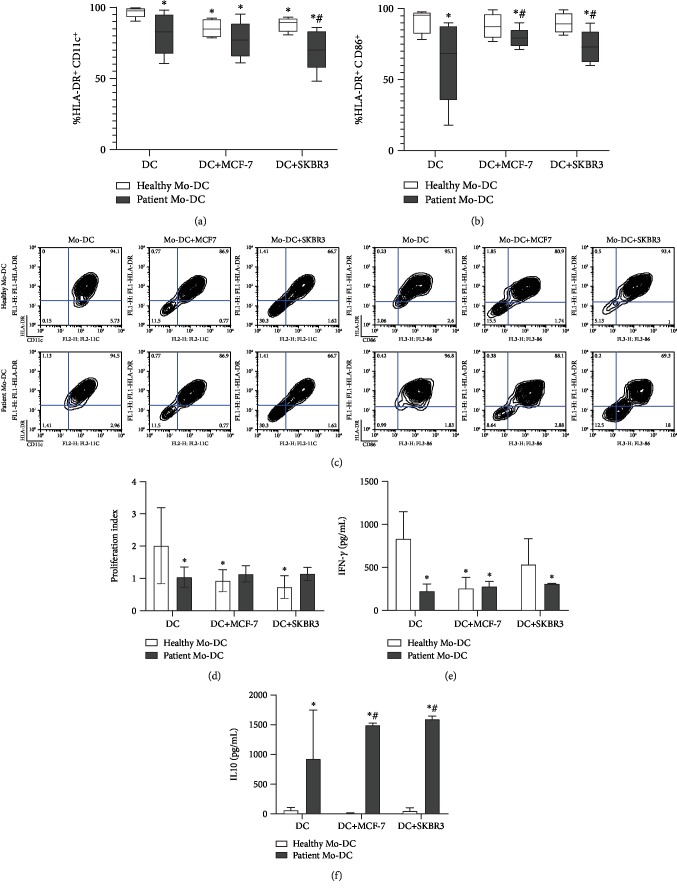
Cancer cell lines (MCF-7 and SKBR3) disturbed the maturation of Mo-DCs. (a, b) Box plots represent the frequency of HLA-DR and CD11c or HLA-DR and CD86-expressing Mo-DCs from healthy donors and breast cancer patients. Monocytes were cultivated in the presence of GM-CSF and IL-4, and Mo-DCs maturation was induced by TNF-*α*. MCF-7 and SKBR3 breast cancer cell lines were cocultured in a transwell system with the Mo-DCs. On day 7 of culture, Mo-DCs were harvested and labelled with monoclonal antibodies for CD11c, CD86, and HLA-DR. (c) Contour plots illustrating HLA − DR × CD11c or HLA − DR × CD86 expression under the various conditions. (d) Proliferation index obtained in mixed lymphocyte reaction (MLR) with healthy donors' or breast cancer patients' Mo-DC cocultured or not with tumor cell lines, as stimulators. (e, f) IFN-*γ* and IL-10 concentrations in MLR supernatants. Cytokine concentrations were determined by ELISA. The results are expressed as the average concentration (pg/mL) ± SEM. (*n* = 4 patients and *n* = 4 healthy donors). A *t*-test was used to compare different groups. ^∗^*p* < 0.05 when compared with Mo-DC obtained from healthy donors and ^#^*p* < 0.05 when compared with Mo-DC from healthy donors in transwell with the respective tumor line cell.

Thus, we evaluated the influence of tumor cells upon healthy donors' Mo-DC maturation. Two different human breast cancer cell lines, MCF7 and SKBR-3, when present during the Mo-DC differentiation, caused a decrease in the frequency of mature DCs, even after TNF-*α* supplementation (Figures 2(a)–2(c)), reduced the Mo-DCs' ability to induce lymphoproliferation (Figure 2(d)), and decreased the IFN-*γ* secretion in the cocultures (Figure 2(e)). Contrastingly, Mo-DCs obtained from patients were not affected, neither on their surface (Figures 2(a)–2(c)) nor on their functional phenotype (Figures 2(d)–2(f)) when exposed to the tumor cells.

**Figure 3:**
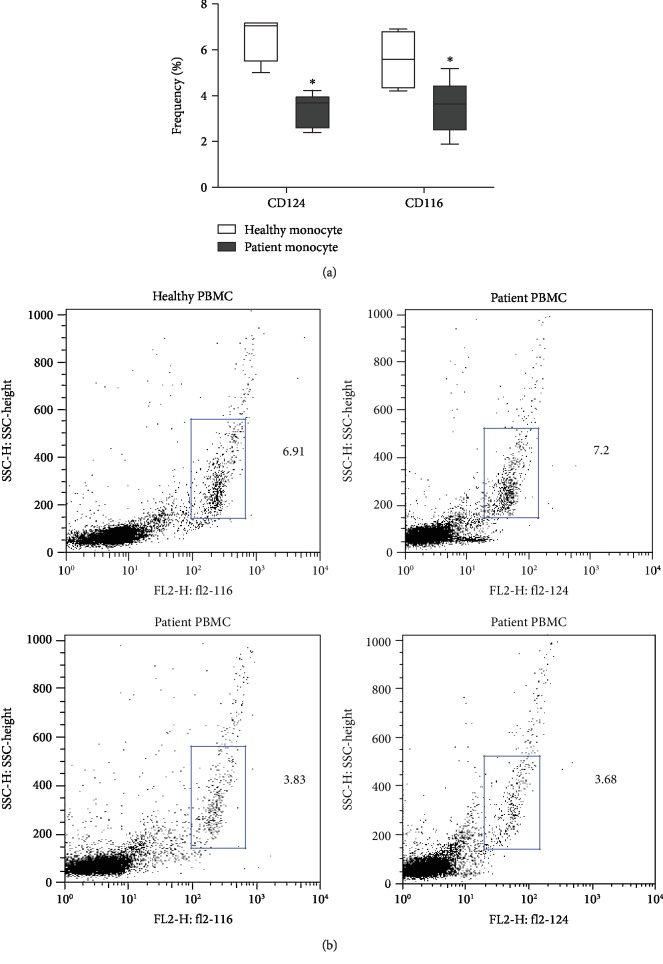
Monocytes from breast cancer patients express less IL-4 and GM-CSF receptors. (a) Box plots representing the frequency of CD124 and CD116 expression by peripheral blood mononuclear cells (PBMCs) obtained from healthy donors and breast cancer patients. (b) Dot plots illustrating the expression of CD124 and CD116 by PBMCs from healthy donors and breast cancer patients.

The combination of granulocyte-macrophage colony stimulating factor (GM-CSF) and interleukin-4 (IL-4) induces the differentiation of Mo-DCs from adherent peripheral blood leukocytes. In accordance with their poorer differentiation into Mo-DCs, peripheral blood mononuclear cells obtained from breast cancer patients showed lower levels of GM-CSF (CD116) and IL-4 receptor (CD124) expression than healthy donors' cells (Figures 3(a) and 3(b)).

**Figure 4:**
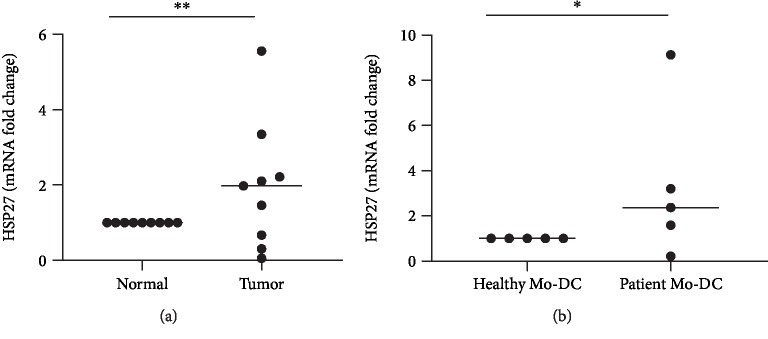
Breast cancer and Mo-DCs obtained from patients express higher levels of HSP27 than healthy cells. (a) Comparative real-time PCR Hsp27 gene expression analysis by tumors (*n* = 9) and normal breast (pooled tissues from 5 donors) shows a significantly (1.46-5.45 fold) increased expression of Hsp27 by tumors. (b) The same analysis performed on Mo-DCs from patients and healthy donors detected an increased Hsp27 expression also by patients' Mo-DCs. GAPDH or *β*-actin were used as internal controls for targeting mRNA expression in tumor and immune cells, respectively. The data were normalized to GAPDH or *β*-actin mRNA expression level. Individual data are plotted, and the horizontal bar represents the mean. Data were compared suing the unpaired *t*-test; ^∗^*p* < 0.05; ^∗∗^*p* < 0.01.

Hsp27 is an immunomodulatory protein expressed by breast cancer cells. In this study, the Hsp27 expression was determined in tumors, normal breast tissue, and Mo-DCs from healthy donors and from patients by quantitative RT-PCR, and a significantly higher Hsp27 expression in tumors (Figure 4(a)) and in Mo-DCs (Figure 4(b)) from patients was detected.

## 4. Discussion

In this study, we investigated the influence of the tumor, *in vivo*, and of tumor cells, *in vitro*, on Mo-DCs. We analyzed their surface phenotype, cytokine secretion, and lymphostimulatory activity and noted that cells from patients or those from healthy donors exposed to tumor cells presented characteristics that could be ascribed to cells with a tolerogenic function. Furthermore, we also noticed the association of these traits with an increased expression of the anti-inflammatory chaperone Hsp27 by Mo-DCs from the patients.

During Mo-DC generation, monocytes were cultured in medium containing GM-CSF and IL-4, cytokines that drive their differentiation into immature DCs, after 5days. After that, TNF-*α* was added as a maturation stimulus. Compared to healthy donor Mo-DCs, breast cancer patient Mo-DCs showed a surface phenotype suggestive of a poorer ability to induce T cell activation and a cytokine profile that, likewise, would drive the induction of tolerance rather than response to the antigens they would present. This bias was maintained even after TNF stimulation, which is known to drive DC maturation and enhance their antigen-presenting function [17, 18]. Within the tumor, abnormal differentiation may generate defective DCs which can contribute to the tumor escape from immune system response [19]. Phenotypic and functional impairment of DCs derived from breast cancer patient monocytes was described in previous studies [20, 21]. The relationship between a cancer immunoenvironment and inflammation is widely accepted. Previous studies showed that tumors induce tolerogenic DCs and, consequently, decrease effective immune responses, thus allowing tumor growth [6, 22, 23]. In breast cancer, a deficiency in mature DC in patients was closely associated with the stage and duration of the disease [24].

The interaction between immune cells and tumor has been investigated by several studies, paving the way for new treatments/therapeutic strategies and indicating new targets for therapy. Using breast cancer cell lines in coculture with monocyte-derived dendritic cells, we obtained results suggesting that the tumor microenvironment not only is able to block DC maturation but also appears to have a systemic effect, preventing the normal differentiation of DC, at least from blood monocytes, thus affecting these central antigen-presenting cells, even before any maturation stimulus. The tumor immunoenvironment is complex and dynamic, with various components, among which is a highly heterogeneous population of inflammatory myeloid cells that may support tumor growth and protect the tumor from host immunity [25]. Tumor progression causes tissue remodeling, metabolic alterations, angiogenesis, changes in tissue cytokine milieu, and cell recruitment, leading to a state similar to that of chronic inflammation [26, 27]. In parallel, clinical and experimental reports indicate that such a proinflammatory microenvironment favors myeloid suppressor cell tumor infiltration and progression [27–31].

The frequency of mature DCs obtained from the differentiation of blood monocytes from breast cancer patients, when compared to healthy donors, was lower. Since GM-CSF and IL-4 are the cytokines used to induce this differentiation [32–34], we investigated the expression of receptors to both cytokines in peripheral blood mononuclear cells from patients and healthy donors. The results showed a loss in both cytokines' receptor expressions in patients' PBMCs. In the inflammatory response, the cytokine signal transduction and the regulation of cytokine gene expression are controlled by heat shock proteins (Hsps) [35]. Hsp27, an important member of the small Hsp family, has been investigated for its elevated intracellular expression and its role as a marker of increased malignancy in human breast tumor cells [13, 36, 37]. Banerjee et al. reported elevated serum Hsp27 levels in breast cancer patients which, in turn, seems to bias the differentiation of monocytes to tolerogenic macrophages, with less tumoricidal activity and with a high proangiogenic capacity that promotes tumor growth [15]. Laudanski et al. demonstrated that Hsp27 treatment reduced DC differentiation levels in IL-4 and GM-CSF-stimulated monocyte cultures [10]. Hsp27 expression was, therefore, investigated, and the results showed a higher expression in breast cancer tissue when compared to normal breast tissue. As mentioned before, Hsp27 has been shown to be upregulated in breast cancer [38], associated with drug resistance [36] and described as able to drive immune cells towards tolerance [10, 15]. Our findings are, accordingly, in agreement with these data and suggest that Hsp27 expression not only by tumor cells but also by antigen-presenting cells may contribute to the immune escape mechanism in breast cancer, favoring the differentiation of DCs biased to induce tolerance rather than response.

DCs are highly specialized antigen-presentation cells that have an important role in the initiation and control of adaptive immunity, determining the immunogenic or tolerogenic response [1, 2, 10, 38, 39]. Le Naour et al. described the gene expression and proteomic profiling changes in Mo-DC differentiation/maturation and reported that during DC differentiation the Hsp27 expression was maximal in immature DCs [40]. Furthermore, exogenous human Hsp27 treatment stimulates monocytes to produce IL-10 and reduces the TNF-*α* levels [41]. Accordingly, we observed that breast cancer and Mo-DCs from breast cancer patients expressed more Hsp27 when compared to nontumor tissues or healthy donor Mo-DCs. Thus, the immature phenotype and regulatory activity that we observed in Mo-DCs from breast cancer patients could be partially caused by the elevated Hsp27 expression within the tumor microenvironment both by the tumor cells and, possibly, by the local DCs (since, at least the Mo-DCs from patients do show this high expression).

## 5. Conclusions

Our study shows that an elevated Hsp 27 expression, already described in breast cancer, is also present in Mo-DCs derived from patients' monocytes. These cells have a surface and functional phenotype that may be indicative of a tolerogenic bias, which could favor tumor escape and growth. Furthermore, we identify a lower expression of GM-CSF and IL-4 receptors by patients' monocytes, suggesting that the interaction of these cytokines and the cells may be less than optimal when patients' cells are involved, a phenomenon that could contribute to their affected phenotype and, likewise, to tumor escape. Though we were not able to causally link the lower expression of cytokines' receptors to the higher Hsp27 expression, it is possible to envisage how such interactions within the complex tumor immunoenvironment could lead an immunosuppressive network that would promote tumor growth and which, therefore, could be possible targets for therapeutic modulation.

## Data Availability

All data that support the manuscript can be accessed in this article and do not have data restriction.
